# Sex-specific molecular signatures in persistent pain after whiplash injury: an exploratory analysis

**DOI:** 10.1097/PR9.0000000000001479

**Published:** 2026-07-31

**Authors:** Joel Fundaun, Colette Ridehalgh, Andrew Dilley, Annina B. Schmid, Georgios Baskozos

**Affiliations:** a Nuffield Department of Clinical Neurosciences, University of Oxford, Oxford, United Kingdom; b Department of Anesthesiology, Perioperative and Pain Medicine, Stanford University School of Medicine, Palo Alto, CA, USA; c Department of Clinical Neuroscience, Brighton and Sussex Medical School, Trafford Centre, University of Sussex, Falmer, Brighton, United Kingdom; d School of Life Course and Population Sciences, King's College London, London, United Kingdom

**Keywords:** Whiplash-associated disorders, RNA-sequencing, Chronic pain, Neuropathic pain, Transcriptional profiling

## Abstract

**Introduction:**

Whiplash-associated disorder grade II (WADII) is characterised by persistent pain in the absence of frank nerve injury, yet its molecular mechanisms remain unclear.

**Objectives:**

To identify molecular signatures of 2 recovery groups characterised by persistent moderate/severe and minimal symptoms in WADII using RNA-sequencing of blood samples 6 months postinjury.

**Methods:**

We performed bulk transcriptional analyses on blood samples collected from the primary cohort of individuals with WADII 6 months postinjury (n = 15/recovery group). Findings were replicated through meta-analyses using an independent cohort of chronic WAD. Blood cellular composition was estimated via deconvolution analyses across both cohorts.

**Results:**

Although there were no differentially expressed genes between recovery groups, we identified sex-specific gene expression signatures of recovery. In the primary WADII cohort, HLA-DQA1, HEBP1, and NECTIN2 showed lower expression in females with moderate/severe symptoms compared with minimal symptoms. SLC12A1 and MXRA7 showed lower expression in males with moderate/severe pain, whereas HLA-G was upregulated. Gene expression meta-analysis in females identified 6 differentially expressed genes, including replication of lower HLA-DQA1 expression from the primary cohort. There were no differentially expressed genes in the meta-analyses in males or between recovery groups. Deconvolution analyses revealed diverging trends between sexes for blood cell types and whiplash symptom severity scores. Ligand–receptor pair analyses further suggested distinct sex-specific recovery trajectories.

**Conclusion:**

Transcriptional profiling revealed sex-specific molecular blood signatures associated with persistent whiplash symptoms 6 months postinjury. Future research is needed to further characterise sex-specific mechanisms after whiplash injury to improve prognosis and targeted management strategies.

## Abstract 2

Supplemental Digital Content is Available in the Text.

Whole-blood RNA-sequencing 6 months after whiplash-associated disorder grade II injury, with independent-cohort meta-analysis, identified sex-specific transcriptional signatures and immune-signalling patterns associated with persistent symptoms.

## 1. Introduction

Persistent pain affects approximately 50% of all individuals with whiplash-associated disorders (WAD) after motor vehicle collisions.^7,55^ Heterogenous clinical presentations and broad classification criteria limit mechanistic understanding of persistent pain in WAD. The standard grading system categorizes WAD severity from 0 to IV; WADII is most common and defined by pain and musculoskeletal dysfunction without frank neurological signs. However, increasing evidence suggests that a spectrum of signs and symptoms are present within these broad classifications.^12,14,16,44^ Our previous prospective cohort study identified signs of nerve pathology and neuropathic pain in over one-third of individuals with WADII 6 months after whiplash.^17,41,42^

There are numerous potential mechanisms underlying persistent pain in WAD, including those related to posttraumatic stress^6,25,27^ and neuroimmune alterations.^39^ Specifically, a recent study in participants with WAD and mild traumatic brain injury (mTBI) 6 months postinjury identified higher expression of SAMD15 mRNA in mTBI participants whose pain resolved.^39^ Although this study highlights circulating immune-related gene expression in recovery, it focused on a more severe injury phenotype involving mTBI. Additional transcriptomic analyses in a less severe and more prevalent injury may help clarify other biological pathways linked to recovery.

Recovery in WAD also exhibits sex-dependent variability; females often demonstrate higher levels of persistent pain compared with males,^4,8,19^ although sex is an inconsistent prognostic factor.^49,55^ Sex-stratified analyses of psychosocial variables, pain-related disability, and somatosensory hypersensitivity demonstrate conflicting results in WAD.^20,22,32,46^ A small number of studies have evaluated sex-specific biological mechanisms of persistent pain in acute WAD using RNA and microRNA expression. Although blood expression levels of microRNA-19b, 17β-estradiol, and μ-opioid receptor 1 allele measured within 24 hours of injury were associated with persistent musculoskeletal after whiplash pain,^26^ our understanding of molecular changes in more chronic phases is currently incomplete.

Our study aimed to identify molecular signatures associated with recovery after whiplash injury in a deeply phenotyped WADII cohort using bulk RNA-sequencing from blood samples collected 6 months after injury. We first evaluated WADII participants by recovery group (minimal vs moderate/severe whiplash symptoms) and then conducted sex-stratified analyses within recovery groups. Our objectives were to (1) evaluate differential gene expression by recovery group and sex; (2) assess replication of transcriptional findings via both a combined differential expression (DE) analysis and meta-analyses with an independent WAD cohort tested at the same postinjury time point; (3) examine associations between gene expression and clinical phenotypes; and (4) evaluate differences in blood cellular composition and ligand–receptor signalling pathways.

## 2. Methods

### 2.1. Participants in the primary cohort

This is a secondary analysis of a previously published, preregistered, prospective cohort study in participants with WADII (ClinicalTrials.gov, version V2 25 September 2020).^17,41–43^ We received ethical approval from the South Central–Oxford C Ethics Committee (18/SC/0263) and London-Brighton & Sussex Research Ethics Committee (20/PR/0625). In brief, participants ranging from 18 to 85 years after motor vehicle collision with a clinical diagnosis of WADII (reduced movements and neck pain without frank neurological signs) were recruited from emergency departments surrounding Brighton and Oxford, United Kingdom. Exclusion included pregnancy; history of cervical/arm pain lasting >3 months; pain from a previous whiplash injury within the previous 12 months; diagnosis of a peripheral neuropathy; and history of systemic illness known to cause small fibre pathology or neuropathy (eg, diabetic neuropathy). Whiplash-associated disorder grade II participants were assessed at a baseline research appointment (<1 month since their motor vehicle crash) and were invited for a follow-up appointment 6 months after their injury date.

### 2.2. Classification of whiplash recovery groups

To assess transcriptional changes associated with persistent whiplash symptoms, we defined recovery groups were defined 6-month postinjury using whiplash symptoms (0–100 visual analogue scale [VAS]): minimal symptoms (VAS < 30/100) and moderate/severe symptoms (VAS ≥ 30/100), using the same strategy as originally performed in the replication cohort.^39^ Based on the previously published replication cohort,^39^ our a priori sample size determined that n = 15 WADII participants/group were needed yielding 80% power to detect 56 prognostic genes out of 10,000 (assuming minimum fold change log2 = 2.0, false discovery rate (FDR) 5%, 100 reads per prognostic gene, and dispersion of 0.16). Sample size was calculated using RnaSeqSampleSize.^57^ After stratification of the WADII cohort into recovery groups, participants were randomly selected for inclusion, with groups matched by age.

### 2.3. Clinical phenotyping

This study reports a subset of the previously published clinical phenotyping measures for the 30 participants with RNA-sequencing selected from the primary cohort.^17,42,43^ Reported demographic details included age, sex, and body mass index (BMI). Phenotypic symptoms from WADII participants at 6-month follow-up included the intensity of neck-related disability using the Neck Disability Index (NDI)^53^; severity of whiplash symptoms (VAS, 0–100); likelihood of neuropathic pain using the painDETECT questionnaire^15^; and the Neuropathic Pain Grading System.^13^ Psychological questionnaires included the posttraumatic stress symptoms from the Impact of Events Scale–Revised,^3^ Pain Catastrophizing Scale,^37^ and Depression, Anxiety, and Stress Scale.^29^ Self-reported whiplash-related treatments at 6-month follow-up included categories for medication, physiotherapy, osteopathy/chiropractic, injections, and other.

### 2.4. RNA extraction

At the 6-month follow-up, blood for RNA-sequencing was collected from the cubital fossa using Tempus Blood RNA Tubes (ThermoFisher, num.4342792,  Waltham, MA). Samples were stored at −20°C until processing. RNA was extracted using the Tempus Spin RNA Isolation Kit (ThermoFisher, num.4380204, Hemel Hempstead, United Kingdom). Blood samples were thawed and diluted with 1x Phosphate-Buffered Saline, vortexed, and centrifuged. We then removed the supernatant and resuspended the RNA pellet. The resuspended RNA was purified and eluted using a microcentrifuge. Extracted RNA was stored at −80°C in Nucleic Acid Purification Solution until sequencing.

### 2.5. RNA-sequencing

RNA-sequencing for the primary cohort was completed by Azenta Life Sciences (Essex, United Kingdom). Library preparation of mRNA used Poly(A) selection. Sequencing was performed in multiplexed lanes using Illumina NovaSeq with paired-end 2 × 150 bp long reads, at a sequencing depth of 20M read pairs per sample. All samples were assessed for RNA degradation and passed initial QC with a RIN > 8.

FastQC^2^/MultiQC and Samtools^23^ were used for quality control of the raw sequencing reads and yield (mean = 28.2 [SD = 5.75] million read pairs), per sequence/per base Phred quality scores (all samples averaged Phred > 35 with no reduction at read extremities), GC content, length distribution, sequence counts, and adapter content. An over-representation of adapter sequences was identified, so reads were trimmed for Illumina adapters using Trimmomatic.^5^ Reads were mapped to the GRCh38 human genome using the STAR aligner^10^ with standard ENCODE parameters. The percentage of uniquely mapped reads was excellent (median = 91.75, interquartile range [IQR] = 1.8), and Cook distance analysis revealed no sample outliers (Supplemental Figure 1, http://links.lww.com/PR9/A432).

### 2.6. Differential expression

We first quantified gene expression in the primary cohort by counting the overlaps between mapped reads^10^ and ENSEMBL gene set annotation GRC.h.38.88 using HTSeq.^1^ Raw sequencing counts were normalised for the effective library size using DESeq2.^28^ We filtered genes to retain those with >20 counts in ≥50% of samples. A negative binomial distribution was fitted to normalised read counts and a Wald test determined DE between recovery groups (minimal vs moderate/severe persistent symptoms), controlling for sex in the primary cohort. Independent gene filtering was done using standard DESeq2 parameters. Normalised counts were log2 transformed for visualisation and correlation analysis purposes. Sex-stratified analyses were performed separately for males and females comparing minimal vs moderate/severe persistent symptoms. These analyses used the same filtering criteria and statistical parameters as the combined analysis. We defined DE genes as those with an FDR < 0.05 and absolute log2 fold change >1.

We performed DE analysis using the same filtering approach and models in the primary cohort alone and the primary and replication cohorts combined while controlling for batch effects and meta-analysed the primary and replication cohorts together. This was done in a sex-stratified way, using an additive blocking design for sex.

### 2.7. Meta-analysis with replication cohort

As a blocking design combining both datasets did not reveal DE genes, meta-analysis was performed to combine statistical evidence across cohorts, increasing sensitivity to detect genes with consistent direction of effect. For this analysis, we used RNA-sequencing data from a previously published cohort postmotor vehicle collision with pain after mTBI (ages 18–70),^39^ referred to as the replication cohort. Blood samples were collected 6 months postinjury, and participants were classified using the same recovery criteria as the primary cohort.^39^ Between-study heterogeneity was quantified using I^2^ (metafor package).

To enable meta-analysis, the replication cohort was reanalysed with the same DE parameters as applied to the primary cohort. This included the same gene filtering criteria (>20 counts in ≥50% samples), controlling for sex, using Wald test without beta prior, and significance thresholds (FDR < 0.05, absolute log2 fold change >1). The SAMD15 associations previously reported 6 months postinjury was not detected, as the model was intentionally designed to match our primary cohort design rather than replicate the original longitudinal parameters (∼sex + age + time + group + time × group).^39^ Sex-stratified analyses were performed using the same parameters controlling for age. Only genes present in both datasets were retained for meta-analysis.

Meta-analysis was performed combining DE results from both cohorts using the R package metaRNAseq.^40^ The inverse normal method was used to combine *P*-values, with filtering for genes showing consistent direction of effect (same sign of log2 fold change) across cohorts. The meta-analysis included the combined primary and replication cohorts controlling for age and sex, as well as a separate sex-stratified meta-analysis controlling for age only.

### 2.8. Cellular deconvolution

The relative abundance of blood cell types was determined using CIBERSORTx.^36^ The LM22 microarray dataset was used to sort immune cell types in the blood.^35^ As data were available in both cohorts, this was done using the combined gene expression data from both the primary and replication cohorts.

### 2.9. Ligand–receptor interaction analysis

We used BulkSignalR^54^ to identify significant ligand–receptor interactions from the merged bulk gene expression data using both the primary and replication cohorts. In brief, BulkSignalR decomposes bulk gene expression data into ligand–receptor pathway triples, identifying significant interactions based on correlated expression patterns across samples rather than DE. Essentially, the correlation between known LR interactors was compared against the baseline correlation of random pairs. BulkSignalR detects correlations between ligands and receptor expression levels, infers networks from these data, and compares the observed correlations with an empirical null distribution derived from random pairs. Information to downstream pathways is derived from numerous databases, including KEGG, Gene Ontology Biological Processes, and Reactome pathway (Pathway Commons v19).^47^ Target genes are identified based on their reachability from the receptor within the pathway network. We analysed ligand–receptor pathway differences by recovery group followed by sex-stratified recovery analyses, using a significance threshold set at a q-value <0.01.

### 2.10. Statistical analyses

Data were analysed using R software (version 4.3.2). The normality of clinical phenotypic data was assessed using visual inspection and the Shapiro–Wilk test. The distribution of phenotypic data was calculated as mean/SD or median/IQR for parametric and nonparametric data, respectively. Two-sided *t* tests or Wilcoxon rank sum tests were used to compare differences in clinical phenotypic data between minimal and moderate/severe recovery groups. A two-sided Fisher exact test was used to compare the distribution of neuropathic pain (NeuPSIG grading) and reported treatments between groups.

We used Spearman rank correlation coefficients to assess the strength of correlation between expression levels of significantly DE genes that survived meta-analyses and phenotypic measures from the primary cohort only, as similar phenotypic data were not available in the replication cohort. For deconvolution analyses, we tested differences in blood cell–type proportions between recovery groups using merged deconvolution data from the primary and replication cohorts, applying a 2-sided Wilcoxon rank-sum test, since both cohorts were generated and analysed using the same pipeline. For sex-stratified blood cell–type analyses, we fit linear regression models including a sex × blood cell–type interaction term to test whether associations between blood cell–type proportions and pain scores differed by sex. We then used Spearman rank correlation to quantify associations between pain scores and inferred blood cell–type proportions separately in males and females. Cell types were preselected for further visualisation if the maximum absolute correlation for either males or females met a threshold of ∣r∣ ≥ 0.30. As the sex-stratified correlation analyses were exploratory in nature, we did not correct for multiple testing. Statistical significance was set at a nominal *P* < 0.05.

### 2.11. Data availability

The RNA-seq data generated during this study, alongside sample group definitions, are available, under managed access, in EGA with accession ID EGAS50000001963.

## 3. Results

Demographic details, including age, sex, and BMI, were similar between recovery groups within the primary cohort (Table 1). Median NDI, whiplash symptoms, and PainDETECT scores were significantly higher in the moderate/severe group compared with minimal (*P* < 0.001). Whiplash-associated disorder grade II participants in the moderate/severe group had significantly higher rates of neuropathic pain according to NeuPSIG classification (*P* = 0.002): 53.5% were classified as probable, 13.3% as possible, and 33.3% as unlikely. In contrast, the minimal symptom group was predominantly classified as unlikely (93.3%), with only one (6.7%) rated as probable. The moderate/severe symptom group had higher posttraumatic stress (*P* = 0.016) and pain catastrophizing (*P* < 0.001) compared with minimal, but no differences in depression, anxiety, or stress (*P* ≥ 0.01). The replication cohort included 36 participants after motor vehicle collision with mTBI (22 males, 14 females; mean age 38.8 years).^39^ Of these, 23 were classified as persistent pain (15 males, 8 females) and 13 as resolved (7 males, 6 females) using identical VAS thresholds (<30 minimal, ≥30 moderate/severe; Supplemental Table 1, http://links.lww.com/PR9/A432).

**Table 1:** Primary cohort participant demographic and symptom characteristics 6 months after whiplash-associated disorder grade II injury.

	Moderate/severe symptoms	Minimal symptoms	*P*
No. of participants	15	15	
Sex (n female, % total)	8 (53.3%)	7 (46.7%)	
Age (median/IQR)	33 (30)	34 (22.5)	0.87
BMI (median/IQR, kg/m^2^)	26.1 (4.87)	25.0 (3.98)	0.50
Follow-up NDI (median/IQR)	13 (6.5)	2 (3)	<0.001
Whiplash symptoms at follow-up (VAS/100; median/IQR)	40 (15.3)	5 (9.5)	<0.001
PainDETECT (median/IQR)*	10.5 (3.8)	2 (6)	<0.001
NeuPSIG grading (%, total)			
Unlikely	33.3% (5/15)	93.3% (14/15)	0.002
Possible	13.3% (2/15)	0% (0/15)	
Probable	53.3% (8/15)	6.7% (1/15)	
Impact of events–revised	23 (31.2)	5 (14)	0.016
Pain catastrophizing scale	20.5 (14.8)	3 (7)	<0.001
DASS			
Depression	8 (10.5)	1 (6.5)	0.10
Anxiety	8 (8)	2.5 (8)	0.13
Stress	14 (11.5)	7 (11.8)	0.11
Treatment (n, %)			
Physiotherapy	7 (50.0%)	6 (40.0%)	1
Osteopathy/chiropractic	3 (21.4%)	0 (0.0%)	0.157
Medication	8 (57.1%)	1 (6.7%)	0.008
Injections	0 (0.0%)	0 (0.0%)	NA
NA other	0 (0.0%)	0 (0.0%)	NA

Follow-up questionnaires were taken 6 months after whiplash injury.

* Values missing for N = 1 participant with moderate/severe symptoms and N = 1 missing with minimal symptoms.

BMI, body mass index; DASS, depression, anxiety, and stress scale; IQR, interquartile range; NDI, neck disability index; NeuPSIG, neuropathic pain special interest group; VAS, visual analogue scale.

### 3.1. RNA-sequencing of blood samples reveals sex-specific transcriptional signatures of whiplash recovery 6 months postinjury

There were no DE genes comparing minimal and moderate/severe pain groups in the primary cohort, controlling for age and sex, in an additive design (Supplemental Figure 2, http://links.lww.com/PR9/A432). Differential expression analysis of the replication cohort alone, and the merged primary and replication cohorts, yielded no significant DE genes (Supplemental Figs. 4–5, http://links.lww.com/PR9/A432). Principal component analysis revealed that sex explained a larger proportion of the variance in gene expression than whiplash symptom severity (Supplemental Figure 3, http://links.lww.com/PR9/A432), supporting further sex-stratified analysis.

**Figure 1.**
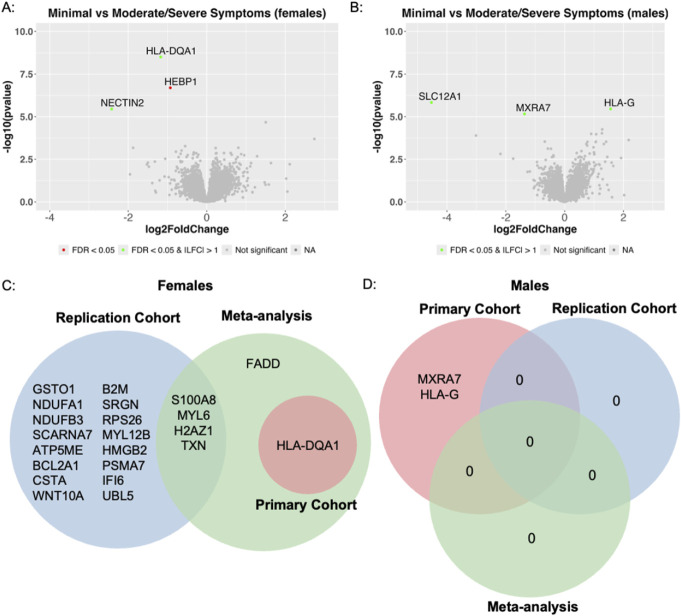
Sex-stratified differential gene expression of the primary cohort by recovery group with meta-analysis with the replication whiplash cohort. (A–B) Differential gene expression comparing minimal vs moderate/severe persistent symptoms recovery groups 6 months after whiplash injury in primary cohort for (A) females and (B) males. (C–D) Gene expression meta-analysis results from the primary and replication cohorts analysed separately for (C) females and (D) males. Primary cohort is in red, replication in blue, and combined meta-analysis results in green. FDR, false discovery rate; LFC, log fold change.

Sex-stratified DE in females revealed 3 genes with lower expression (HLA-DQA1, HEBP1, and NECTIN2) in moderate/severe compared with minimal symptoms (Fig. 1A). In males, SLC12A1 and MXRA7 had lower expression in the moderate/severe group, whereas HLA-G had higher expression compared with males with minimal symptoms (Fig. 1B).

### 3.2. Gene expression meta-analyses identify sex-specific profiles and replicate HLA-DQA1 lower expression in females

Gene expression meta-analysis did not identify any DE genes between minimal and moderate/severe groups from the combined primary and replication WAD cohorts. Female meta-analysis identified 6 DE genes with high between-study heterogeneity (I^2^ = 84.7%, Fig. 1C): HLA-DQA1, which was significantly DE in the primary cohort alone, 4 genes (S100A8, MYL6, H2AZ1, TXN) that were significantly DE in the replication cohort alone, and one gene identified from the meta-analysis (FADD) not seen in the individual cohort analyses. However, there were no DE genes that survived meta-analysis within the male group (Fig. 1D).

### 3.3. Targeted genes from meta-analyses reveal diverging correlations with neuropathic pain, whiplash symptoms, and neck-related disability

**Figure 2.**
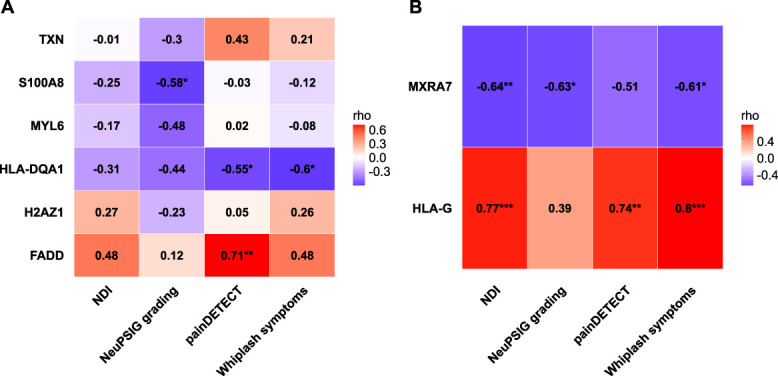
Correlation of pain and disability phenotype information. (A–B) Correlation plots for the genes identified after meta-analysis and pain/disability phenotypic data in the primary cohort for (A) females and (B) males. *P < 0.05; **P < 0.01; ***P < 0.001.

In females from the primary cohort, HLA-DQA1 expression showed a moderate negative correlation with whiplash symptom severity (r = −0.60, *P* = 0.004) and painDETECT (r = −0.60, *P* = 0.033; Fig. 2A, Supplemental Table 2, http://links.lww.com/PR9/A432). FADD was positively correlated with painDETECT scores (r = 0.71, *P* = 0.003), and S100A8 was negatively correlated with NeuPSIG grading (r = −0.58, *P* = 0.028) in females from the primary cohort. There were no significant associations between H2AZ1, MYL6, or TXN with any pain, symptom, or disability measure (*P* > 0.05). In males, HLA-G gene expression showed a strong positive correlation between NDI (r = 0.77 *P* = 0.001), PainDETECT (r = 0.74, *P* = 0.003), and whiplash symptom severity (r = 0.80, *P* < 0.001; Fig. 2B, Supplemental Table 2, http://links.lww.com/PR9/A432).

### 3.4. Deconvolution analyses reveal sex-dependent correlations of whiplash symptoms and blood cell types

**Figure 3.**
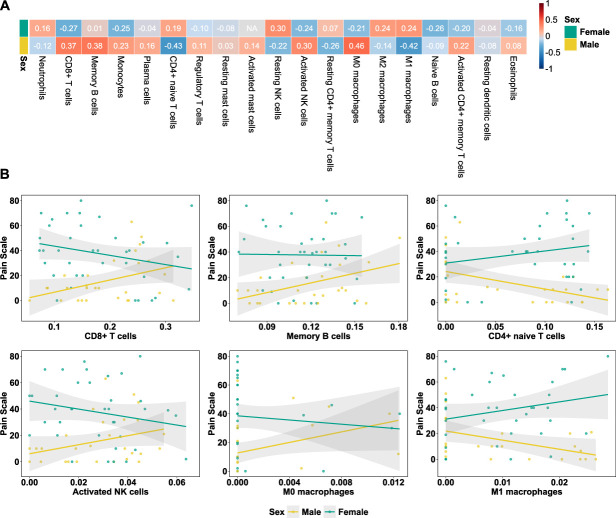
Sex-based differences in blood cell types and whiplash symptoms. (A) Sex-specific correlation heatmap illustrating relationships between selected blood cell types and whiplash symptoms, across merged data from both cohorts. (B) Sex-stratified correlations between selected blood cell types, restricted to cell types with |r| ≥ 0.30 in either sex (males, yellow; females, green). Linear trend lines for visualization.

There were no significant differences in blood cell types between WAD recovery groups using merged primary and replication cohorts (*P* > 0.212, Supplemental Figure 6, http://links.lww.com/PR9/A432, Supplemental Table 3, http://links.lww.com/PR9/A432). Sex-stratified interaction analyses suggested the association between proportions of blood cell types and whiplash symptoms differed by sex for CD8^+^ T cells (*P* = 0.02), CD4^+^ naïve T cells (*P* = 0.01), resting natural killer cells (*P* = 0.03), monocytes (*P* = 0.02), and M1 macrophages (*P* = 0.02, Supplemental Table 4, http://links.lww.com/PR9/A432). Exploratory correlation analyses identified sex-specific directional patterns in the proportion of blood cell types and whiplash symptom severity between sexes (Fig. 3A). Examples of diverging correlations are shown in Figure 3B and Supplemental Table 5, http://links.lww.com/PR9/A432.

### 3.5. Ligand–receptor pathway analyses indicate sex-stratified immune signalling

**Figure 4.**
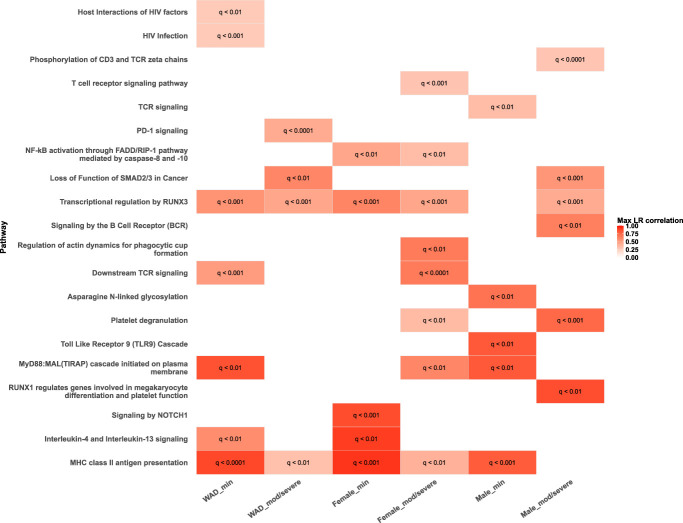
Pathway-level ligand–receptor interaction strength by recovery status and sex. Heatmaps illustrate the maximum correlation among ligand–receptor pairs maintained after FDR q < 0.01 for each group and pathway. Data include combined primary and replication cohorts. WAD minimal symptoms (n = 28), WAD mod/severe symptoms (n = 38), female minimal symptoms (n = 13), female mod/severe symptoms (n = 16), male minimal symptoms (n = 15), and male mod/severe symptoms (n = 22). LR, ligand–receptor; min, minimal; mod, moderate; WAD, whiplash-associated disorders.

In participants with minimal symptoms, the most strongly correlated pathways included major histocompatibility complex (MHC) class II antigen presentation (q < 0.0001), MyD88:MAL(TIRAP) signalling (q < 0.01), and TLR9 cascade (q < 0.01, Fig. 4; Supplemental Table 6, http://links.lww.com/PR9/A432). In the moderate/severe symptom WAD group, transcriptional regulation by RUNX3 (q < 0.01) and PD-1 signalling (q < 0.001) were most strongly correlated. In sex-stratified analyses, females with minimal symptoms showed the strongest correlations for MHC class II antigen presentation (q < 0.0001), followed by IL-4/IL-13 (q < 0.01) and NOTCH1 signalling (q < 0.01). In females with moderate/severe symptoms, downstream TCR signalling was strongest (q < 0.0001). Males with minimal symptoms demonstrated strongest correlations in MyD88:MAL (TIRAP) and TLR9 pathways (q < 0.01), whereas males with moderate/severe symptoms were strongest for RUNX1-regulated megakaryocyte differentiation and platelet function (q < 0.01) and platelet degranulation (q < 0.001).

## 4. Discussion

Approximately 50% of individuals post-WADII experience persistent pain, yet the underlying molecular mechanisms remain unclear. Our whole-blood transcriptional profiling did not identify any DE genes between WADII recovery groups 6 months postinjury. However, we did identify clear sex-specific gene expression signatures of recovery. Using meta-analyses with an independent whiplash cohort, we replicated the female-specific lower expression of HLA-DQA1, which negatively correlated with whiplash symptom severity and painDETECT scores. In males, HLA-G showed higher expression with moderate/severe symptoms in the primary cohort and positively correlated with whiplash symptom severity, painDETECT, and neck-related disability scores, although not replicated in the male meta-analysis. Blood cellular deconvolution and ligand–receptor interaction analyses further suggested distinct sex-specific mechanisms of whiplash recovery. Taken together, our results demonstrate sex-specific molecular signatures of whiplash recovery 6 months postinjury.

### 4.1. Lower expression of HLA-DQA1 expression and major histocompatibility complex class II antigen presentation pathways correlate with persistent whiplash symptoms in females 6 months postinjury

In both the primary and replication cohorts, MHC class II gene HLA-DQA1 had lower expression in females with moderate/severe symptoms 6 months after whiplash injury. The HLA system is critical for immune recognition and antigen presentation^9^ and is one of the most polymorphic regions in the human genome.^51^ Our sex-specific gene expression patterns of whiplash recovery are supported by a recent study that showed a similar decrease in HLA-DQA1/DQB in peripheral blood leukocytes in females and an increase in males after injection of the immune stimulant, lipopolysaccharide.^50^

In females, reduced HLA-DQA1 expression and sex-stratified CD8^+^ T-cell interactions suggest one potential adaptive immune mechanism underlying persistent whiplash symptoms. Reduced HLA-DQ protein expression has been directly linked to CD8^+^ T-cell suppression. Salgame et al. showed that HLA-DQ protein presentation, and not HLA-DR or HLA-DP subclasses, was critical for CD8^+^ T suppressor cell function in human T-cell clones derived from peripheral blood.^48^ Reduced HLA-DQA1 expression may, therefore, contribute to the sex-divergent trends. CD8^+^ T cells in our data showed a trend for a negative correlation with whiplash symptom severity in females, whereas males demonstrated positive associations, albeit not statistically significant.

Our ligand–receptor analyses further support potential coordinated changes in T-cell receptor signalling and antigen presentation pathways in females with persistent whiplash symptoms. We identified enriched pathways in T-cell receptor signalling (B2M-CD3G, B2M-CD247) and MHC class II antigen presentation (APP–CD74). Our meta-analyses identified additional genes implicated in T-cell function, including FADD, TXN, H2AZ1, and S100A8, which are associated with T-cell memory, homeostasis, signalling attenuation, and proliferation.^24,30,31,34^ Among these genes, FADD positively correlated with painDETECT scores, whereas S100A8 negatively correlated with NeuPSIG grading in females 6 months after whiplash injury. Collectively, these findings point to a sex-specific adaptive immune response related to the reduced HLA-DQA1 gene expression and MHC class II antigen presentation in females with persistent whiplash symptoms.

### 4.2. HLA-DQA1 expression is linked to neuropathic pain phenotypes after whiplash injury

HLA-DQA1 has been strongly linked with neuropathic pain, particularly after traumatic nerve injuries. The largest genome-wide association study of neuropathic pain to date identified HLA-DQA2 as a significant locus associated with central neuropathic pain.^38^ Similarly, a previous meta-analysis of genome-wide association studies identified HLA-DQB1*03 polymorphisms, which is a heterodimer with HLA-DQA1 and creates a functional HLA-DQ molecule.^21^ HLA-DQB1*03 carriers had 2.86-fold higher odds of neuropathic pain compared with nonneuropathic controls.^52^ Similarly, recent evidence identified the HLA A02:01-B15:01-C03:04-DRB104:01-DQB1*03:02 haplotype with 9.3-fold higher odds of having neuropathic pain after nerve injury postsurgery compared with nonneuropathic nerve injury controls.^33^

Decreased HLA-DQA1 expression in our study may, therefore, reflect a transcriptional signature of chronic neuropathic pain more broadly, rather than a process specific to whiplash injuries. In our larger cohort study, two-thirds of WADII participants were classified as having at least possible neuropathic pain acutely after whiplash injury, and one-third had persisting neuropathic pain 6 months postinjury.^17,41^ We also identified signs of nerve injury and inflammation in up to 40% of WADII participants using clinical neurological assessments, quantitative sensory testing, magnetic resonance neurography of the brachial plexus and dorsal root ganglia, and skin biopsies at 6-month follow-up.^17,41,42^ Consistent with these findings, signs of neural injury were also present in the replication cohort, which included participants presenting with acute mTBI after whiplash injury.^39^ Further investigation using combined genotyping and expression analyses is needed to confirm whether lower expression of HLA-DQA1 reflects functional changes in neuropathic pain–associated risk alleles.

### 4.3. Higher expression of HLA-G and platelet degranulation correlate with persistent whiplash symptoms in males 6 months postinjury

HLA-G had higher expression in males with moderate/severe vs minimal symptoms at 6 months, although this finding was not replicated in the meta-analysis. HLA-G expression also positively correlated with whiplash symptoms, painDETECT, and neck-related disability scores. This pattern aligns with a broader immune signature in males after whiplash, characterised by ligand–receptor pathways enriched for platelet degranulation.

Although evidence of its involvement in persistent pain is limited, HLA-G protein levels were positively correlated with platelet counts in patients with immune thrombocytopenia (an autoimmune condition characterized by reduced platelet counts) and healthy controls.^56^ Taken together, our findings suggest an ongoing inflammatory phenotype, including active adaptive immune response and platelet degranulation, may contribute to persistent symptoms in males after whiplash injury. Further characterization is needed to clarify how HLA-G may contribute to persistent whiplash symptoms.

### 4.4. Clinical considerations

Although recovery differences were not detectable in combined-sex analyses, the sex-stratified findings represent an important development in understanding immune-mediated mechanisms of whiplash recovery. Immune dysregulation has previously been identified after whiplash injuries, as evidenced by acute and chronically altered blood inflammatory mediators^11,42^ and peripheral neuroinflammation of the brachial plexus and dorsal root ganglia detected through increased T2 signal on magnetic resonance imaging.^17,18^ Our sex-specific HLA-related gene expression results build on this work by providing novel biological evidence for distinct immune-mediated mechanisms underlying chronic whiplash symptoms. Future studies should evaluate sex-stratified immunomodulatory treatment in whiplash. Prognostically, HLA-expression may compliment current survey-based tools predicting whiplash outcomes (eg, Whip Predict).^45^ Prospective longitudinal validation in larger cohorts, however, is required to evaluate its clinical utility.

### 4.5. Strengths and limitations

A strength of our study is the replication of gene expression using meta-analyses with an independent whiplash cohort. However, the cross-sectional design prevents a complete understanding how transcriptional changes occur over time. The lack of detailed medication reports may also underestimate transcriptional effects. Although we were not fully powered for sex-stratified analyses within the primary cohort, our replication in an independent cohort and formal meta-analysis were used to strengthen inference and identify genes with consistent effects across datasets. Although the correlation between gene expression levels and pain scores is compelling, further confirmation in larger cohorts is needed to understand the unique contribution of HLA dysregulation to recovery after whiplash injury and to neuropathic pain more broadly. Future studies should also use single-cell RNA-sequencing to evaluate specific blood cell–type contributions to the immune signatures identified here.

## 5. Conclusions

We demonstrate divergent DE patterns of whiplash recovery, with lower expression of HLA-DQA1 in females and higher expression of HLA-G in males 6 months postinjury. These molecular patterns are further supported by sex-dependent blood cell and ligand–receptor pathway analyses. Our findings underscore the importance of evaluating sex-based differences in persistent symptoms after whiplash injury to develop improved treatment strategies. Further mechanistic investigation into sex-specific HLA-subtype and immune profiles in WAD recovery are warranted to advance personalised pain management for whiplash injuries.

## Disclosures

C.R. and J.F. are supported by a Versus Arthritis Pain Challenge Grant (22465). J.F. is also supported by an NIH National Institute of Drug Abuse T32DA035165 grant, NIHR Oxford Biomedical Research Centre Doctoral Award, and Annabel Foundation. G.B. is funded by Diabetes UK (19/0005984). A.B.S. is supported by a Wellcome Trust Clinical Career Development Fellowship (222101/Z/20/Z) and the Medical Research Foundation (Emerging Leaders Prize in Pain Research). G.B. and A.B.S. are members of the PAINSTORM consortium as part of the Advanced Pain Discovery Platform (MR/W002388/1) supported by the Medical Research Council and Versus Arthritis. A.D. has no conflicts of interest.

## Supplemental digital content

Supplemental digital content associated with this article can be found online at http://links.lww.com/PR9/A432.
